# A Technological Review of the Instrumented Footwear for Rehabilitation with a Focus on Parkinson’s Disease Patients

**DOI:** 10.3389/fneur.2016.00001

**Published:** 2016-01-20

**Authors:** Justyna Maculewicz, Lise Busk Kofoed, Stefania Serafin

**Affiliations:** ^1^Sound and Music Computing Group, Department of Architecture, Design and Media Technology, Aalborg University Copenhagen, Copenhagen, Denmark

**Keywords:** instrumented footwear, rhythmic rehabilitation, Parkinson’s disease, gait rehabilitation, auditory feedback, haptic feedback

## Abstract

In this review article, we summarize systems for gait rehabilitation based on instrumented footwear and present a context of their usage in Parkinson’s disease (PD) patients’ auditory and haptic rehabilitation. We focus on the needs of PD patients, but since only a few systems were made with this purpose, we go through several applications used in different scenarios when gait detection and rehabilitation are considered. We present developments of the designs, possible improvements, and software challenges and requirements. We conclude that in order to build successful systems for PD patients’ gait rehabilitation, technological solutions from several studies have to be applied and combined with knowledge from auditory and haptic cueing.

## 1 Introduction

The purpose of this review article is to present a context of usage of instrumented footwear in Parkinson’s disease (PD) patients’ auditory and haptic rehabilitation. We present developments of the design, possible improvements, and software challenges and requirements. We summarize existing technological solutions and applications. Literature on rhythmic auditory rehabilitation provides design requirements for the hardware and software, which is necessary to build successful rehabilitation wireless systems for PD patients with instrumented footwear as a data collector and feedback device.

## 2 A Context of Interactive Shoe Usage

Interactive shoes with embedded sensors have been used in many different scenarios. Gait analysis is the prominent one. Tao et al. (1) reviewed gait analysis challenges and presented a selection of wearable sensors, which can be used for gait analysis. In the context of PD, it is necessary to collect information about patients’ balance (2), gait cadence, velocity, and stride length (3). Interactive shoes give opportunities for collecting data about users’ current state. This can be followed by notifying users or supervising persons (e.g., doctors and physiotherapists). Data gathered by sensors can send information to rehabilitation systems when a specific problem or need occur and activate cueing stimuli in the form of auditory signals or vibrations.

### 2.1 Gait Impairments in PD

Gait impairments have received a lot of attention in recent years since they are a common cause of disability in people with PD (4). Various aspects of gait have been found to be affected by PD, but they can be influenced and improved through rehabilitation based on auditory or haptic cueing. The most common ones are freeze of gait (5), balance (6), gait velocity, cadence, stride length (3), increased spatio-temporal variability (7), and difficulties with gait initiation (8). These disturbances are lead to restricted mobility, weakened balance, and consequently to increased risk of falling (9). Such disturbances have been found to influence patients’ general quality of life (10).

### 2.2 Rehabilitation through Auditory Stimulation

#### 2.2.1 Metronome-Like Rhythmic Stimulation

It has been shown that following a rhythmic auditory cue helps gait performance in patients with PD (11–15). The PD patients are usually provided with an auditory metronome, or markedly rhythmic music, and asked to match consecutive footfalls with the onset of each beat (16). External rhythms presented by auditory cues may improve gait characteristics (13–15) and can also be used to identify deficits in gait adaptability (17). Spaulding et al. (3) in their review pointed that the auditory cueing elicited positive changes in gait cadence, velocity, and stride length.

#### 2.2.2 Mutual Entrainment

The aforementioned way of stimulation lacks the interactivity component where the system could adapt to the user. In this case, mutual entrainment between system and user happens. Miyake (18) proposed the Walk-Mate to implement the mutual entrainment for the rehabilitation of PD and hemiplegic patients. Baram (19) suggested that gait rehabilitation must be performed in a closed-loop system to avoid constant vigilance and need of attention strategies to prevent reversion to impaired gait patterns caused by repetitive stimuli. Hove et al. (20) reported that random, disconnected stride times (low fractal scaling) predicts falling for PD patients. Fixed rhythmic auditory stimulation lowers fractal scaling and requires attention. Gait rehabilitation should lead to achieving the more stable and not random stride times structure, which can be observed in healthy gait (20). Systems based on mutual entrainment principles can emergently respond to unpredictable changes in human behavior (21). The studies by Hove et al. (20) and Uchitomi et al. (22) showed that the gait fluctuation of the patients gradually returned to a healthy stride times fluctuation level in the interactive conditions. This effect did not occur in fixed tempo and no-cue conditions.

#### 2.2.3 Improvements through Ecological Stimuli

Rhythmic sounds (metronome-like) only specify step duration of gait, with no information relating to spatio-temporal properties of walking actions. Rodger et al. (16) recently proposed the use of ecological signals as a new approach to auditory rehabilitation. Ecological signals, defined as those stimuli, which are encountered in everyday life, have the potential to convey richer information. Listeners, based on footstep sounds, can determine gender and mood of the walking person (23). Complex walking sounds, such as footsteps on gravel, may convey both temporal (step duration) and spatial (step length) properties of gait (16).

### 2.3 The Advantages of Haptic Stimulation

Little research has focused on foot-based vibrotactile systems. The sensitivity of the sensory system of the feet is sufficient for vibrotactile guidance (24). Signals from mechanoreceptors in the foot are one of the main sensory sources for gait generation and modification (25). It is likely that mechanoreceptive afferents in the sural nerve provide rich information about contact patterns between the foot and the environment during stance and locomotion (24). When exposed to audio and haptic stimulation, subjects are able to best recognize different materials delivered haptically or as a combination of auditory and haptic feedback (26). Both auditory and haptic feedback are represented as temporal variations, which can be simulated with similar patterns, at different frequency ranges. Since most of the pedestrians wear shoes when walking it makes it an excellent platform for mounting actuators (27).

## 3 The Existing Technologies

This section presents studies describing foot plantar measurement systems and their usage in auditory and haptic rehabilitation and focuses on main advantages coming from their possible usage.

### 3.1 Foot Plantar Measurement Systems

In this subsection, we will mention a few interesting instrumented insoles which followed by a review by Abdul Razak et al. (28).

Amjadi et al. (29) presented a flexible foot pad containing force-sensitive resistors arrays for the foot sole distributed force detection. Stassi et al. (30) described an easy and cost-effective approach used to fabricate the conformable insole based on a piezoresistive material. It measures both the pressure distribution under 64 nodes arranged in the main plantar regions and the mean plantar pressure during walking activity with a sampling frequency of 20 Hz. While developing instrumented insoles, Tamm et al. (31) focused mostly on accuracy, long-term stability and reproducibility, and time resolution. They proposed a thin, light weight self-contained platform for mobile wireless pressure sensing insole system with 24 separated points of measurement on the foot. Suresh et al. (32) demonstrated a proof-of-concept of a new high-resolution plantar pressure monitoring pad based on fiber Bragg grating (FBG) sensors. Motha et al. (33) introduced a unique approach to measure applied pressure. The change in capacitance is entirely led by variation of relative permittivity of the surrounding dielectric medium with applied pressure. Tan et al. (34) presented another low-cost design for plantar pressure measurements. They proposed a system based on carbon-embedded piezoresistive material sandwiched between two layers of electrodes to form a pressure sensing insole.

### 3.2 Foot Plantar Measurement Systems with Its Tested Applications

Redd and Bamberg (35) presented a simple system of two force-resistive sensors per insole, connected to a mobile application that delivers feedback. Their tests showed that the feedback system is capable of influencing the gait of the user, without the need for direct supervision by a rehabilitation specialist. The system proposed by Santoso and Setyanto (36) consists of a sensing unit and a signal processing unit. The sensing unit is based on piezoelectric stress sensor module (two in each insole) and data acquisition module both wirelessly connected. The system is able to differentiate running from walking in athletes performance. Madavi and Giripunje (37) proposed a wearable device for the diabetic person of sensory neuropathy. It identifies the ulcerous condition, which may be created in foot plantar surface area. Temperature sensors or pressure sensors are used to detect the infected area. The output data can be transmitted wirelessly to the hospital system. Grenez et al. (38) described the development of a hardware system simulating a shoe, which consists of three pressure sensors, two bending sensors, an accelerometer, an Arduino mini, and a Bluetooth module. The developed prototype is able to differentiate between healthy gait and imperfect gait. Holleczek et al. (39) described the development of textile pressure sensors, which are more comfortable in usage than standard ones. The textile pressure sensors were developed using the principle of a variable capacitor. These sensors were attached to the socks (three in each sock at relevant positions under the heel and the ball of the foot) of a snowboarder for the monitoring of the in-shoe pressure distribution.

### 3.3 Instrumented Footwear Systems with Vibrating Stimulation

Hijmans et al. (40) described a technology, which could be used in the future to improve balance in healthy young and older people and in patients with a stroke or diabetic neuropathy. The system uses cork insole covered with a leather layer. A C2 electromechanical actuator and a piezo actuator or the VBW32 skin transducer, activated by a custom-made noise generator, were chosen to provide tactile stimulation to the feet.

The goal of the application called Gilded Gait is to simulate the perception of a range of different ground textures and serve as the navigation in the city (41). The system contains six vibrations panels to present the feedback patterns, push-down switch, and an accelerometer to detect user’s steps. Three different patterns of vibrations were designed to simulate different ground textures. Recognition of the patterns was possible only if they were asked to choose from the list rather than recall a material.

Velázquez et al. (42) described the development of a tactile communication system (16 actuators embed into a shoe insole) and a pilot study on recognition of different information (direction, pattern, emotion recognition, and language learning) assigned arbitrary to vibration patterns, which as was shown can be easily learnt and understood.

### 3.4 A System Based on Accelerometer

Walk-Mate (43) is a system mainly used as a gait compensation device and as a gait rehabilitation training device by analyzing improvements in locomotion before, during, and after rehabilitation in hemiparetic patients and comparing it with a previous gait training method. Walk-Mate generates a model walking rhythm in response to the user’s locomotion in real time, and by indicating this rhythm using auditory stimuli, provides a technology that supports walking by reducing asymmetries and fluctuations in foot contact rhythm.

### 3.5 More Complex Systems

Watanabe and Ando (27) introduced a system called Pace-synch shoes. Pressure sensors embedded in a shoe sole served as step detectors and provided data for a vibration motor to be activated. The users reported that when the vibration was presented at heel-strike timing, it was perceived as natural, while the vibrations at the other timings caused odd feelings. They also reported that, when they walked matching their step cycles to the vibration at the heel-strike timing, their way of walking did not subjectively change. As the authors claimed, their method would be applicable for training and coaching in sports and for rehabilitation in health care.

The system described in Zanotto et al. (6) allows for synthesizing continuous audio-tactile feedback in real time, based on the readings of piezoresistive and inertial sensors embedded in the footwear. The system contains 4 piezoresistive sensors, a 9-degree-of-freedom inertial measurement unit, and five actuators in each shoe. All information is stored and processed in the belt unit. The results of this preliminary experiment indicated that ecological underfoot feedback may alter the natural gait pattern of healthy subjects.

The prototype proposed by Tajadura-Jiménez et al. (44) allows for the dynamic modification of footstep sounds, as people walk, and measures changes in walking behavior. This sandals-based system captures sounds of a person’s footsteps via a microphone attached to the sandals. Two force-sensitive resistors are attached to the front and the rear part of the sandal insole that detect the exerted force by feet against the ground as well. A triple axis accelerometer was attached to the walker’s left ankle. Augmenting the high frequencies of the sound leads to the perception of having a thinner body and enhances the motivation for physical activity inducing a more dynamic swing and a shorter heel strike.

### 3.6 Existing Application for PD Patients

The work presented by Winfree et al. (45) is one of the most interesting studies in the context of this review. These authors described a prototype of a shoe-based system, which contains FSR sensors and an actuator, which are activated in the certain situations. The system was used in a short intervention study with PD patients. The most crucial aspects of the system are its portability, wireless communication, low-cost development, adjustable automatic software, ease of learning, and presentation of appropriate audio and haptic signals. The core of the system operates in a way that if only the ball or toe of the foot is in contact with the ground, the toe actuator vibrates. When both the heel and ball or toe are concurrently in contact, both tactors vibrate. This condition is met during stance phase of ambulation. The Berg Balance Scale (46), Timed Up and Go (47) performance tests, and the FOG questionnaire (48) were used to obtain measurement data during pre-and post-treatment. It was shown that this stimulation provoked significant changes to all measures except time on toe sensor and step duration.

Bächlin et al. (49) aimed to develop a system, which overcomes the limitations of previous systems, such as the continuous nature of the cueing intervention, manually triggered cueing or provided only during training sessions, but not provided at the time of episodic gait disturbances. The challenge set by these authors is to detect freeze of gait episode and apply automatically interactive rhythmic auditory stimulation to overcome the problem. This system consists of a wearable computer, a set of acceleration sensors and headphones. The study was the first one in which FOG is automatically detected, and the results are very promising. The system detected FOG with high sensitivity and also received acceptance from the users.

## 4 Emerging Guidelines for Interactive Shoes Design for PD and Discussion

Identifying non-invasive treatments to alleviate the symptoms of PD is important to improving PD patients’ life quality (45). Several studies exploring rhythmic auditory stimulation (RAS) and its modified interactive versions in PD rehabilitation showed improvements in gait cadence, stride length, and gait velocity (3). It is promising to use all above mentioned knowledge and technology to build an instrumented footwear system for the PD rehabilitation based on data collected from the instrumented footwear and feedback presentation through auditory and tactile channels. This kind of a system gives a lot of opportunities for a remote communication between a patient and a doctor or therapist. System interactivity allows for not only presenting feedback but also giving cues for patients based on their performance. The system could detect the events such as FOG, loss of balance and impaired gait patterns and subsequently present cues to correct it and help patients overcome these issues.

### 4.1 Hardware

Our literature review shows that from the hardware perspective pressure sensors, actuators and accelerometer need to be embedded into the instrumented footwear system. Pressure sensors will allow for step detection, and for monitoring balance, and pressure applied to selected parts of the feet. Important is the choice of the pressure sensors with adequate accuracy and durability. The aforementioned studies exhibit that it is possible to use a wide variety of pressure sensors and materials from which they are made. Moreover, they present high diversity in the number of data collecting points, ranging from 2 (35) to 75 (34). It is possible to find low-cost solutions in both categories. However, the number of the sensors embedded in a shoe sole depends on the available calculating power of each system. These data can be easily used as a basis for calculating velocity, stride length, cadence, and temporal variability.

The actuators present haptic feedback or cues to the users. Haptic feedback has three main advantages: it can be hidden in the shoe, can motivate users to perform a step by detecting FOG or gait initiation, and can increase the perceived naturalness of the auditory stimuli, which can serve as a higher motivation for rehabilitation. There are no sufficient studies to indicate the best placement of actuators in a shoe sole; based on the study by Watanabe and Ando (27), we believe that a heel is the best potential candidate to be stimulated by an actuator. Although Kennedy and Inglis (50) indicated that the ball and the arch are the most sensitive areas to vibrotactile stimulation.

The placement of an accelerometer is quite optional, but it should be hidden in the shoe and well protected from the displacement. Accelerometers collect data about feet acceleration in three-dimensional space. They give more precise information about feet movement than FSR sensors, and they are crucial in detecting balance problems. An accelerometer was successfully used for FOG detection in Bächlin et al. (49).

### 4.2 Software/Application

Few studies consider the use of ecological signals, despite their richness of information and acceptance from the users’ perspective. Bächlin et al. (49) demonstrated the need for a context-aware system. The ideal system should be adaptive to the participant’s speed (19, 20) and able to present cueing signals constantly or in *ad hoc* manner (49). The overall goal is to design a system, which patients would like to wear everyday and feel comfortable with it. For example, auditory cueing should be used by patients only during short sessions every day. The haptic stimulation could serve constantly, especially when a person would like to go out of their home. According to the patients’ needs, it should be able to choose constant or *ad hoc* stimulation by choosing program on the main computer unit. Each system should be personalized and be programed for specific needs such as FOG, loss of balance, or slowed pace, to be mentioned among others.

## 5 Summary

In this review, we summarized systems for gait rehabilitation based on instrumented footwear. We focused on the need of PD patients, but since only a few systems where made with this purpose, we went through several applications used in different scenarios when gait detection and rehabilitation is considered. Future designs could benefit from this knowledge. We outlined the hardware and software needs to run rehabilitation with the use of haptic and auditory cueing and feedback. There is still work to be done, but since technology for foot plantar measurement and feedback presentation is developing very fast, we should focus on specific applications and build customized systems for everyday use. The future trends outlined as well by Bächlin et al. (49) are: (1) miniaturization of the system and the main operating unit, which could be a part of the patients everyday clothing and hidden to make user feel comfortable; (2) specific calibration and customization of the rehabilitation programs based on patient-specific problems; (3) possibility of outdoor usage, so patients will be more secure and independent.

## Author Contributions

JM is the most responsible person for this article. Since the type of article is mini review, she was responsible for literature search and selection and writing part. Both LK and SS were consulted at each step of article preparation, writing and corrections.

## Conflict of Interest Statement

The authors declare that the research was conducted in the absence of any commercial or financial relationships that could be construed as a potential conflict of interest.
